# 2-(4-Fluoro­anilino)-3-(2-hydroxy­ethyl)quinazolin-4(3*H*)-one

**DOI:** 10.1107/S1600536809006254

**Published:** 2009-02-28

**Authors:** Qian Zhang, Yuan-Hong Jiao, Bin Liu, Xue-Mei Chen, Min Ruan, Ling-Hua Xu

**Affiliations:** a School of Chemistry and Material Engineering, Huangshi Institute of Technology, Huangshi 435003, People’s Republic of China

## Abstract

The mol­ecular and crystal structures of the title compound, C_16_H_14_FN_3_O_2_, are stabilized by intra­molecular N—H⋯O and inter­molecular O—H⋯O hydrogen bonds. The existence of non-classical intra­molecular C—H⋯N hydrogen bonds provides a dihedral angle between the fluoro-substituted benzene and pyrimidinone rings of 7.9 (1)°.

## Related literature

For the pharmacological activity of N3 and C7 disubstituted quinazolines, see: Usha *et al.* (2006 ▶). For the synthesis of quinazolinone and thienopyrimidinones, see: Yang *et al.* (2008 ▶). For synthesis, drug discovery and crystal structures, see: Yang & Wu (2008 ▶); Wang *et al.* (2008 ▶).
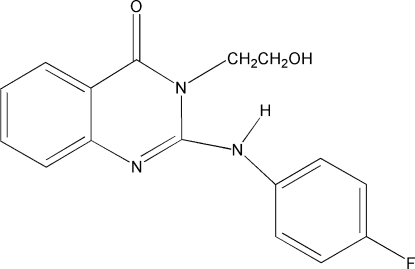


## Experimental

### 

#### Crystal data

C_16_H_14_FN_3_O_2_
*M* *_r_* = 299.30
Monoclinic, 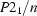

*a* = 8.5737 (8) Å
*b* = 10.8268 (10) Å
*c* = 15.2490 (13) Å
β = 104.070(10)°
*V* = 1371.0 (2) Å^3^
*Z* = 4
Mo *K*α radiation
μ = 0.11 mm^−1^
*T* = 273 K
0.10 × 0.10 × 0.10 mm

#### Data collection

Bruker SMART CCD area-detector diffractometer
Absorption correction: none
8342 measured reflections
2976 independent reflections
2475 reflections with *I* > 2σ(*I*)
*R*_int_ = 0.021

#### Refinement

*R*[*F* ^2^ > 2σ(*F* ^2^)] = 0.037
*wR*(*F* ^2^) = 0.102
*S* = 1.06
2976 reflections
205 parameters
2 restraints
H atoms treated by a mixture of independent and constrained refinement
Δρ_max_ = 0.13 e Å^−3^
Δρ_min_ = −0.24 e Å^−3^

### 

Data collection: *SMART* (Bruker, 2007 ▶); cell refinement: *SAINT* (Bruker, 2007 ▶); data reduction: *SAINT*; program(s) used to solve structure: *SHELXS97* (Sheldrick, 2008 ▶); program(s) used to refine structure: *SHELXL97* (Sheldrick, 2008 ▶); molecular graphics: *SHELXTL* (Sheldrick, 2008 ▶); software used to prepare material for publication: *SHELXTL*.

## Supplementary Material

Crystal structure: contains datablocks I, global. DOI: 10.1107/S1600536809006254/rk2120sup1.cif

Structure factors: contains datablocks I. DOI: 10.1107/S1600536809006254/rk2120Isup2.hkl

Additional supplementary materials:  crystallographic information; 3D view; checkCIF report

## Figures and Tables

**Table 1:** Hydrogen-bond geometry (Å, °)

*D*—H⋯*A*	*D*—H	H⋯*A*	*D*⋯*A*	*D*—H⋯*A*
C5—H5⋯N3	0.93	2.28	2.8807 (16)	122
N1—H1⋯O2	0.881 (9)	1.982 (10)	2.8253 (14)	159.7 (13)
O2—H2*A*⋯O1^i^	0.837 (9)	1.909 (10)	2.7426 (13)	173.6 (18)

Symmetry code: (i) 
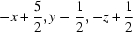
.

**Fig. 1.**
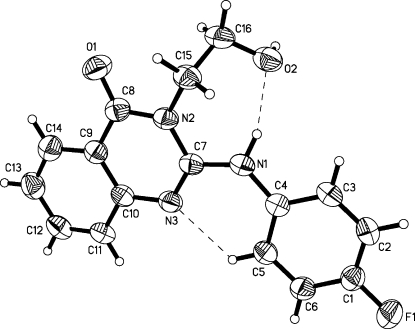
View of the title molecule with the atom–labeling scheme. Dispalcement ellipsoids are drawn at the 50% probability level. H at6oms are presented as a small spheres of arbitrary radius. Intramolecular hydrogen bonds are drawn by dashed lines.

**Fig. 2.**
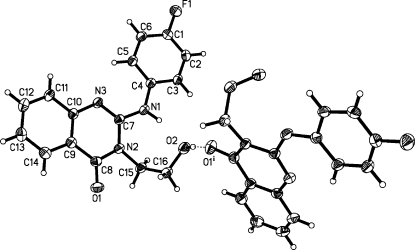
Part of the crystal structure of the title compound showing intermolecular hydrogen bonds as dashed lines. Symmetry code: (i) -x+5/2, y-1/2, -z+1/2.

## supplementary crystallographic information

### Comment

Quinazolone derivatives have evoked considerable attention in recent years as these are endowed with wide range of pharmaceutical activities. 3*H*–Quinazolin–4–one represents a useful nucleus for preparation of some new sedative/hypnotic and anticonvulsant agents. Of the various quinazolines reported, the N3 and C7 disubstituted quinazolines exhibit interesting pharmacological activities like analgesic, anti–inflammatory, antibacterial and anticonvulsant activities (Usha *et al.*, 2006). In connection with our ongoing heterocyclic synthesis and drug discovery project (Yang & Wu, 2008), we have focused on the synthesis of quinazolinone and thienopyrimidinones (Wang *et al.*, 2008; Yang *et al.*, 2008). Herein, the title compound was synthesied and its molecular (Fig. 1) and crystal structures were determined. An intramolecular N1—H1···O2 and non–classical C5—H5···N3 hydrogen bonds provide the conformation of the molecule - dihedral angle between the fluoro–substituted benzene ring and pyrimidinone rings as 7.9 (1)°. In the crystal structure, intermolecular O2—H2a···O1^i^ hydrogen bonds link of molecules into chains (see Tab. 1 and Fig. 2). Symmetry code: (i) -x+5/2, y-1/2, -z+1/2.

### Experimental

To a solution of 2–ethoxycarbonyliminophosphorane (1.27 g, 3.0 mmol) in 10 ml anhydrous *THF*, 4–fluorophenyl isocyanate (0.41 g, 3.0 mmol) was added dropwise at room temperature. The reaction mixture was left unstirred for 6 h at 273–278 K, whereafter the above resulting solution was added dropwise to a solution of ethanolamine (0.18 g, 3 mmol) in 5 ml anhydrous *THF*. The reaction mixture was stirred overnight at room temperature, the solvent was removed under reduced pressure and the residue was recrystallized from C_2_H_4_Cl_2_/CH_3_OH (1:1/v:v) to give colourless crystals of the title compound. Yield 89%.

### Refinement

The C–bonded H atoms were placed in calculated positions with C—H = 0.93 Å for aromatic, C—H = 0.97 Å for methylene and refined in the riding model approximation. The positional parameters of N– and O–bonded H atoms were found from difference Fourier map and refined independently. For all H atoms *U*_iso_(H) = 1.2*U*_eq_(C, N) and *U*_iso_(H) = 1.5*U*_eq_(O).

### Crystal data

**Table:**

C_16_H_14_FN_3_O_2_	*F*(000) = 624
*M**_r_* = 299.30	*D*_x_ = 1.450 Mg m^−^^3^
Monoclinic, *P*2_1_/*n*	Mo *K*α radiation, λ = 0.71073 Å
Hall symbol: -P 2yn	Cell parameters from 3320 reflections
*a* = 8.5737 (8) Å	θ = 2.3–29.2°
*b* = 10.8268 (10) Å	µ = 0.11 mm^−^^1^
*c* = 15.2490 (13) Å	*T* = 273 K
β = 104.407 (1)°	Block, colourless
*V* = 1371.0 (2) Å^3^	0.10 × 0.10 × 0.10 mm
*Z* = 4	

### Data collection

**Table:**

Bruker SMART CCD area-detector diffractometer	2475 reflections with *I* > 2σ(*I*)
Radiation source: Fine–focus sealed tube	*R*_int_ = 0.021
Graphite	θ_max_ = 27.0°, θ_min_ = 2.3°
φ and ω scans	*h* = −10→10
8342 measured reflections	*k* = −12→13
2976 independent reflections	*l* = −19→14

### Refinement

**Table:**

Refinement on *F*^2^	Primary atom site location: Direct
Least-squares matrix: Full	Secondary atom site location: Difmap
*R*[*F*^2^ > 2σ(*F*^2^)] = 0.037	Hydrogen site location: Geom
*wR*(*F*^2^) = 0.102	H atoms treated by a mixture of independent and constrained refinement
*S* = 1.06	*w* = 1/[σ^2^(*F*_o_^2^) + (0.0546*P*)^2^ + 0.157*P*] where *P* = (*F*_o_^2^ + 2*F*_c_^2^)/3
2976 reflections	(Δ/σ)_max_ = 0.001
205 parameters	Δρ_max_ = 0.13 e Å^−^^3^
2 restraints	Δρ_min_ = −0.23 e Å^−^^3^

### Special details

**Table:**

Geometry. All s.u.'s (except the s.u. in the dihedral angle between two l.s. planes) are estimated using the full covariance matrix. The cell s.u.'s are taken into account individually in the estimation of s.u.'s in distances, angles and torsion angles; correlations between s.u.'s in cell parameters are only used when they are defined by crystal symmetry. An approximate (isotropic) treatment of cell s.u.'s is used for estimating s.u.'s involving l.s. planes.
Refinement. Refinement of *F*^2^ against ALL reflections. The weighted *R*–factor *wR* and goodness of fit *S* are based on *F*^2^, conventional *R*–factors *R* are based on *F*, with *F* set to zero for negative *F*^2^. The threshold expression of *F*^2^ > σ(*F*^2^) is used only for calculating *R*–factors(gt) *etc*. and is not relevant to the choice of reflections for refinement. *R*–factors based on *F*^2^ are statistically about twice as large as those based on *F*, and *R*–factors based on ALL data will be even larger.

### Fractional atomic coordinates and isotropic or equivalent isotropic displacement parameters (Å^2^)

**Table:**

	*x*	*y*	*z*	*U*_iso_*/*U*_eq_	
C1	0.56770 (14)	0.34529 (11)	−0.09177 (9)	0.0442 (3)	
C2	0.59291 (15)	0.34162 (11)	0.00070 (9)	0.0455 (3)	
H2	0.5444	0.2813	0.0283	0.055*	
C3	0.69136 (15)	0.42908 (11)	0.05135 (8)	0.0427 (3)	
H3	0.7096	0.4276	0.1141	0.051*	
C4	0.76459 (13)	0.52003 (10)	0.01077 (8)	0.0379 (3)	
C5	0.73709 (15)	0.52084 (12)	−0.08302 (8)	0.0438 (3)	
H5	0.7852	0.5807	−0.1113	0.053*	
C6	0.63794 (15)	0.43247 (12)	−0.13419 (8)	0.0458 (3)	
H6	0.6193	0.4325	−0.1969	0.055*	
C7	0.94732 (13)	0.70237 (10)	0.05017 (7)	0.0377 (3)	
C8	1.13407 (14)	0.86392 (11)	0.11798 (8)	0.0418 (3)	
C9	1.15360 (14)	0.88561 (11)	0.02741 (8)	0.0402 (3)	
C10	1.06202 (13)	0.81559 (11)	−0.04382 (8)	0.0389 (3)	
C11	1.08192 (15)	0.83508 (12)	−0.13148 (8)	0.0462 (3)	
H11	1.0220	0.7892	−0.1798	0.055*	
C12	1.18945 (16)	0.92159 (13)	−0.14591 (9)	0.0520 (3)	
H12	1.2025	0.9334	−0.2041	0.062*	
C13	1.27943 (17)	0.99209 (13)	−0.07485 (10)	0.0549 (3)	
H13	1.3514	1.0510	−0.0857	0.066*	
C14	1.26168 (16)	0.97446 (12)	0.01099 (10)	0.0504 (3)	
H14	1.3215	1.0216	0.0586	0.060*	
C15	0.97517 (16)	0.76912 (12)	0.21207 (8)	0.0450 (3)	
H15A	0.8617	0.7484	0.1999	0.054*	
H15B	0.9889	0.8505	0.2395	0.054*	
C16	1.06872 (18)	0.67775 (13)	0.27916 (8)	0.0526 (3)	
H16A	1.1828	0.6861	0.2827	0.063*	
H16B	1.0521	0.6944	0.3387	0.063*	
F1	0.46983 (11)	0.25850 (8)	−0.14287 (6)	0.0651 (3)	
N1	0.85952 (13)	0.60633 (10)	0.07009 (7)	0.0439 (2)	
H1	0.8863 (16)	0.5818 (12)	0.1269 (6)	0.053*	
N2	1.02153 (12)	0.77481 (9)	0.12481 (6)	0.0393 (2)	
N3	0.96041 (12)	0.72240 (9)	−0.03130 (6)	0.0411 (2)	
O1	1.20713 (12)	0.91951 (9)	0.18653 (6)	0.0565 (3)	
O2	1.01772 (12)	0.55593 (9)	0.25230 (6)	0.0546 (3)	
H2A	1.0973 (16)	0.5093 (14)	0.2698 (12)	0.082*	

### Atomic displacement parameters (Å^2^)

**Table:**

	*U*^11^	*U*^22^	*U*^33^	*U*^12^	*U*^13^	*U*^23^
C1	0.0436 (6)	0.0404 (6)	0.0481 (7)	−0.0004 (5)	0.0105 (5)	−0.0022 (5)
C2	0.0492 (7)	0.0396 (6)	0.0504 (7)	0.0018 (5)	0.0174 (6)	0.0082 (5)
C3	0.0499 (7)	0.0442 (6)	0.0356 (6)	0.0072 (5)	0.0135 (5)	0.0067 (5)
C4	0.0379 (6)	0.0404 (6)	0.0360 (6)	0.0046 (5)	0.0104 (5)	0.0012 (5)
C5	0.0492 (7)	0.0480 (7)	0.0357 (6)	−0.0038 (5)	0.0132 (5)	0.0024 (5)
C6	0.0508 (7)	0.0519 (7)	0.0352 (6)	−0.0028 (6)	0.0114 (5)	−0.0017 (5)
C7	0.0370 (6)	0.0411 (6)	0.0333 (6)	0.0052 (5)	0.0058 (4)	0.0015 (5)
C8	0.0416 (6)	0.0426 (6)	0.0409 (6)	0.0040 (5)	0.0094 (5)	−0.0044 (5)
C9	0.0388 (6)	0.0399 (6)	0.0420 (6)	0.0059 (5)	0.0102 (5)	0.0020 (5)
C10	0.0377 (6)	0.0406 (6)	0.0376 (6)	0.0065 (5)	0.0080 (5)	0.0053 (5)
C11	0.0476 (7)	0.0538 (7)	0.0351 (6)	0.0046 (6)	0.0060 (5)	0.0081 (5)
C12	0.0531 (7)	0.0602 (8)	0.0442 (7)	0.0058 (6)	0.0149 (6)	0.0147 (6)
C13	0.0513 (8)	0.0542 (8)	0.0607 (9)	−0.0035 (6)	0.0168 (6)	0.0112 (7)
C14	0.0489 (7)	0.0483 (7)	0.0536 (8)	−0.0022 (6)	0.0121 (6)	−0.0010 (6)
C15	0.0535 (7)	0.0494 (7)	0.0348 (6)	0.0053 (6)	0.0160 (5)	−0.0031 (5)
C16	0.0621 (8)	0.0616 (8)	0.0327 (6)	0.0034 (6)	0.0092 (6)	−0.0009 (6)
F1	0.0736 (6)	0.0599 (5)	0.0592 (5)	−0.0229 (4)	0.0116 (4)	−0.0068 (4)
N1	0.0521 (6)	0.0494 (6)	0.0297 (5)	−0.0036 (5)	0.0092 (4)	0.0026 (4)
N2	0.0434 (5)	0.0436 (5)	0.0316 (5)	0.0037 (4)	0.0104 (4)	−0.0013 (4)
N3	0.0436 (5)	0.0456 (6)	0.0331 (5)	0.0000 (4)	0.0077 (4)	0.0028 (4)
O1	0.0601 (6)	0.0639 (6)	0.0450 (5)	−0.0115 (5)	0.0123 (4)	−0.0152 (4)
O2	0.0629 (6)	0.0552 (6)	0.0421 (5)	0.0091 (4)	0.0062 (4)	0.0055 (4)

### Geometric parameters (Å, °)

**Table:**

C1—C6	1.3659 (17)	C9—C14	1.4007 (17)
C1—F1	1.3670 (14)	C10—N3	1.3771 (15)
C1—C2	1.3729 (18)	C10—C11	1.4050 (16)
C2—C3	1.3716 (18)	C11—C12	1.3699 (19)
C2—H2	0.9300	C11—H11	0.9300
C3—C4	1.3929 (16)	C12—C13	1.391 (2)
C3—H3	0.9300	C12—H12	0.9300
C4—C5	1.3904 (16)	C13—C14	1.3683 (19)
C4—N1	1.4098 (15)	C13—H13	0.9300
C5—C6	1.3846 (17)	C14—H14	0.9300
C5—H5	0.9300	C15—N2	1.4817 (14)
C6—H6	0.9300	C15—C16	1.5035 (18)
C7—N3	1.2932 (14)	C15—H15A	0.9700
C7—N1	1.3616 (15)	C15—H15B	0.9700
C7—N2	1.3983 (15)	C16—O2	1.4174 (17)
C8—O1	1.2335 (14)	C16—H16A	0.9700
C8—N2	1.3866 (15)	C16—H16B	0.9700
C8—C9	1.4511 (16)	N1—H1	0.881 (9)
C9—C10	1.3952 (16)	O2—H2A	0.837 (9)
			
C6—C1—F1	119.04 (11)	C12—C11—H11	120.0
C6—C1—C2	122.08 (12)	C10—C11—H11	120.0
F1—C1—C2	118.88 (11)	C11—C12—C13	120.99 (12)
C3—C2—C1	118.42 (11)	C11—C12—H12	119.5
C3—C2—H2	120.8	C13—C12—H12	119.5
C1—C2—H2	120.8	C14—C13—C12	119.76 (12)
C2—C3—C4	121.34 (11)	C14—C13—H13	120.1
C2—C3—H3	119.3	C12—C13—H13	120.1
C4—C3—H3	119.3	C13—C14—C9	120.19 (13)
C5—C4—C3	118.79 (11)	C13—C14—H14	119.9
C5—C4—N1	125.37 (11)	C9—C14—H14	119.9
C3—C4—N1	115.82 (10)	N2—C15—C16	114.93 (10)
C6—C5—C4	119.90 (11)	N2—C15—H15A	108.5
C6—C5—H5	120.0	C16—C15—H15A	108.5
C4—C5—H5	120.0	N2—C15—H15B	108.5
C1—C6—C5	119.46 (11)	C16—C15—H15B	108.5
C1—C6—H6	120.3	H15A—C15—H15B	107.5
C5—C6—H6	120.3	O2—C16—C15	109.98 (11)
N3—C7—N1	121.83 (11)	O2—C16—H16A	109.7
N3—C7—N2	123.86 (10)	C15—C16—H16A	109.7
N1—C7—N2	114.31 (10)	O2—C16—H16B	109.7
O1—C8—N2	119.47 (11)	C15—C16—H16B	109.7
O1—C8—C9	124.96 (11)	H16A—C16—H16B	108.2
N2—C8—C9	115.56 (10)	C7—N1—C4	128.75 (10)
C10—C9—C14	120.21 (11)	C7—N1—H1	115.3 (9)
C10—C9—C8	118.50 (11)	C4—N1—H1	113.5 (9)
C14—C9—C8	121.29 (11)	C8—N2—C7	120.89 (10)
N3—C10—C9	122.90 (10)	C8—N2—C15	116.33 (10)
N3—C10—C11	118.21 (11)	C7—N2—C15	122.56 (10)
C9—C10—C11	118.75 (11)	C7—N3—C10	117.72 (10)
C12—C11—C10	120.09 (12)	C16—O2—H2A	107.5 (13)
			
C6—C1—C2—C3	0.31 (19)	C12—C13—C14—C9	0.1 (2)
F1—C1—C2—C3	179.97 (10)	C10—C9—C14—C13	−0.76 (19)
C1—C2—C3—C4	0.08 (18)	C8—C9—C14—C13	179.16 (12)
C2—C3—C4—C5	−0.34 (18)	N2—C15—C16—O2	−74.18 (14)
C2—C3—C4—N1	178.41 (11)	N3—C7—N1—C4	−3.84 (19)
C3—C4—C5—C6	0.23 (18)	N2—C7—N1—C4	176.74 (11)
N1—C4—C5—C6	−178.39 (11)	C5—C4—N1—C7	−3.0 (2)
F1—C1—C6—C5	179.92 (11)	C3—C4—N1—C7	178.32 (11)
C2—C1—C6—C5	−0.42 (19)	O1—C8—N2—C7	−174.16 (11)
C4—C5—C6—C1	0.14 (19)	C9—C8—N2—C7	6.98 (16)
O1—C8—C9—C10	−179.80 (11)	O1—C8—N2—C15	11.04 (16)
N2—C8—C9—C10	−1.02 (16)	C9—C8—N2—C15	−167.81 (10)
O1—C8—C9—C14	0.28 (19)	N3—C7—N2—C8	−9.29 (17)
N2—C8—C9—C14	179.06 (11)	N1—C7—N2—C8	170.11 (10)
C14—C9—C10—N3	176.35 (11)	N3—C7—N2—C15	165.17 (11)
C8—C9—C10—N3	−3.57 (17)	N1—C7—N2—C15	−15.43 (15)
C14—C9—C10—C11	0.73 (17)	C16—C15—N2—C8	−94.13 (13)
C8—C9—C10—C11	−179.20 (10)	C16—C15—N2—C7	91.16 (14)
N3—C10—C11—C12	−175.91 (11)	N1—C7—N3—C10	−174.89 (10)
C9—C10—C11—C12	−0.08 (18)	N2—C7—N3—C10	4.47 (17)
C10—C11—C12—C13	−0.6 (2)	C9—C10—N3—C7	1.96 (17)
C11—C12—C13—C14	0.5 (2)	C11—C10—N3—C7	177.61 (10)

### Hydrogen-bond geometry (Å, °)

**Table:**

*D*—H···*A*	*D*—H	H···*A*	*D*···*A*	*D*—H···*A*
C5—H5···N3	0.93	2.28	2.8807 (16)	122
N1—H1···O2	0.88 (1)	1.98 (1)	2.8253 (14)	160 (1)
O2—H2A···O1^i^	0.84 (1)	1.91 (1)	2.7426 (13)	174 (2)

Symmetry codes: (i) −*x*+5/2, *y*−1/2, −*z*+1/2.
